# Acupoint injection of Bacillus Calmette–Guerin polysaccharide nucleic acid for patients with chronic urticaria: A protocol for systematic review

**DOI:** 10.1097/MD.0000000000019924

**Published:** 2020-05-01

**Authors:** Wei Cao, Xianjun Xiao, Leixiao Zhang, Lu Wang, Qianhua Zheng, Siyuan Zhou, Ying Liu, Yue Cao, Mingling Chen, Chunxiao Li, Ying Li

**Affiliations:** a Acupuncture and Tuina School, Chengdu University of Traditional Chinese Medicine; b Rehabilitation Department, The People's Hospital of Jianyang City; c Dermatological Department, Affiliated Hospital of Chengdu University of Traditional Chinese Medicine, Chengdu, Sichuan, China.

**Keywords:** acupoint, acupoint injection, Bacillus Calmette–Guerin, Bacillus Calmette–Guerin polysaccharide nucleic acid, chronic urticaria, protocol, systematic review

## Abstract

**Background:**

To investigate the efficacy and safety of acupoint injection of Bacillus Calmette–Guerin polysaccharide nucleic acid (BCG-PSN) in the treatment of chronic urticaria (CU).

**Methods:**

The following databases will be searched from their inception: Medline, Embase, Pubmed, Web of Science, Cochrane Central Register of Controlled Trials, China National Knowledge Infrastructure Database, China Biomedical Literature Database, China Science Journal Database, and Wanfang Database. All databases will be searched from the date of creation until October 2019. In addition, we will manually search the list of medical journals as a supplement. The scope of the search included randomized controlled clinical studies related to acupoint injection of BCG-PSN for CU. The primary outcome is the disease activity control. Secondary outcomes include response rate, adverse events, and recurrence rates. The Cochrane RevMan V5.3 Deviation Assessment Tool will be used to assess bias assessment risk, data integration risk, meta-analysis risk, and subgroup analysis risk (if conditions are met). The average difference, standard mean difference and binary data will be used to represent continuous results.

**Results:**

This study will comprehensively review the existing evidence on the treatment of CU by acupoint injection of BCG-PSN.

**Conclusion:**

This systematic review will provide a judgment basis for the effectiveness and safety of acupoint injection of BCG-PSN in the treatment of CU.

**Systematic review registration:**

PROSPERO, CRD42019139885.

## 1 Introduction

### 1.1 Description of the condition

Urticaria is defined as the sudden development of transient hives (wheals) and angioedema or both. A wheal is characterized by a circumscribed superficial edema of the skin, mostly surrounded by bright red erythema and associated with a strong itching or burning sensation. Chronic urticarial (CU) is defined as the presence of urticaria for a period exceeding 6 weeks, Attack twice a week or more.^[1,2]^ It is divided into chronic inducible urticarias and chronic spontaneous urticaria.^[3]^ The etiology of CU is still unknown, but many shreds of evidence suggest that it may be related to different biological systems, such as immunity, inflammation, and coagulation, which causes mast cells and basophils to degranulate and form wheal.^[4–7]^ According to the survey, the global incidence of CU is close to 1%.^[8–11]^ It can occur in any age group^[12]^ and has a higher incidence in women than men.^[9,13–15]^ In most cases, symptoms in more than 70% of CU patients will last 2 to 5 years, 20% of them will last more than 5 years, If patients have hypertension, this can be as high as 74%.^[9,16–18]^ 1.8% of CU patients will never recover.^[19]^ Patients have a poor quality of life, with nearly 50% of them suffering from moderate to severe disease activity. CU patients are often accompanied by anxiety, depression, difficulty sleeping, social disorders, and so on, which have a significant impact on life of CU patients.^[13,20–23]^

Based on the consensus^[24,25]^ and the guidelines^[1,26,27]^ published between 2014 and 2018, Second-generation H1 antihistamines (sgAH) are the drugs of choice for initial therapy for CU.^[1]^ If unbearable symptoms appear after 2 to 4 weeks or earlier, the dose of the sgAH can be increased to 4 times the manufacturer's recommended dose (both unlicensed). Omalizumab should be added if it still does not improve significantly. If there is no satisfactory improvement within half a year, it is recommended to use cyclosporine and sgAH for treatment.^[17,27,28]^ The sgAH can improve symptoms, but not reduce the total number of itching wheal episodes.^[29,30]^ Studies have shown that long-term use of antihistamines can cause headaches, drowsiness, fatigue, dry mouth, allergies and other adverse reactions,^[31–33]^ the median tolerance to h1-antihistamines in CU patients was 3 years, and 88.9% of patients had poor CU control.^[34]^ Omalizumab has been shown to be effective,^[35–37]^ but its expensive cost cannot be ignored.^[38]^ Currently, there is no effective treatment for all patients, so it is necessary to explore and evaluate some other treatments to provide more options for the clinical treatment of CU.

### 1.2 Description of the intervention

Acupuncture is an important part of complementary and alternative medicine, Studies have demonstrated its effectiveness and safety in treating CU.^[39–41]^ Acupoint injection is 1 of the treatments of acupuncture which emerged in China during the 1950 s, and it is a modified acupuncture technique.^[42]^ it originated from intra-muscular injection in western medicine and was gradually integrated into traditional Chinese medicine.^[43,44]^ Acupoint injection is an acupoint-stimulating technique in which a liquid agent is injected to prevent and/or treat diseases,^[45]^ The agents generally include Western medications, Chinese herbal extractions, vitamins, bee venom, and normal saline solution. It is widely used in China for CU^[42,46,47]^ and had good curative effects.^[48–50]^

Among then, Bacillus Calmette–Guerin polysaccharide nucleic acid (BCG–PSN) is the most commonly used 1 in China.^[51]^ BCG-PSN is 1 of the Immunomodulators which participate in immunomodulatory actions,^[52]^ It is a mixture of nucleic acids and polysaccharides extracted from BCG immune-active substances. Some studies have shown that BCG-PSN can be used in allergic diseases.^[51,52]^ At present, there are many studies on the treatment of CU by injecting BCG-PSN into acupoints in China, and the results were positive.^[53–56]^ But 1 study has reported that BCG-PSN combined with antihistamine did not increase the effect of treating CU.^[57]^ So far, its clinical evidence is insufficient.

From what has been discussed above, The efficacy and safety of acupoint injection of BCG-PSN in the treatment of CU have not been systematically evaluated. Therefore, it is necessary to make treatment recommendations based on the available evidence.

## 2 Methods and analysis

### 2.1 Study registration

This Study registration is designed in strict compliance with the preferred reporting items for systematic reviews and meta-analysis protocol.^[58]^ and has been registered at PROSPERO (ID: CRD42019139885).

### 2.2 Ethics and Dissemination

The results of this systematic review are to evaluate the efficacy and safety of published randomized controlled clinical trial (RCT) about acupoint injections of BCG-PSN for CU to help clinicians and patients choose the appropriate treatment regimen. This review does not require ethical approval and will be reported in peer-reviewed journals.

### 2.3 Search strategy

We will use computers to search Medline, Embase, Pubmed, Web of Science and Cochrane Central Register of Controlled Trials and China's four databases: China National Knowledge Infrastructure Database, China Biomedical Literature Database, China Science Journal Database, and Wanfang Database. All databases will be searched from the date of creation until October 2019. The following search terms will be used: Urticaria, CU, Nettle-rash, Hives, Rubella, Wind cluster, Angioedema, acupoint injection, acupuncture point injection, acupoint-injection, hydro-acupuncture, point injection, acupoint block, BCG, Bacillus Calmette–Guerin, BCG-PSN, BCG polysaccharide nucleic acid, Bacillus Calmette–Guerin polysaccharide nucleic acid, Boolean operators ‘and’ and ‘or’ will be used within the search to combine the search terms. The example search strategy in Table 1 will be used for Pubmed. The search strategy for each of the other sites is adapted to the characteristics of the database. We will search the list of related references for more trials and existing systematic reviews (SRs) related to our topics by PubMed and Cochrane Library, and will also search a reference list to identify published journals related to the research topic, Books, conference articles, and grey literature.

**Table 1:**
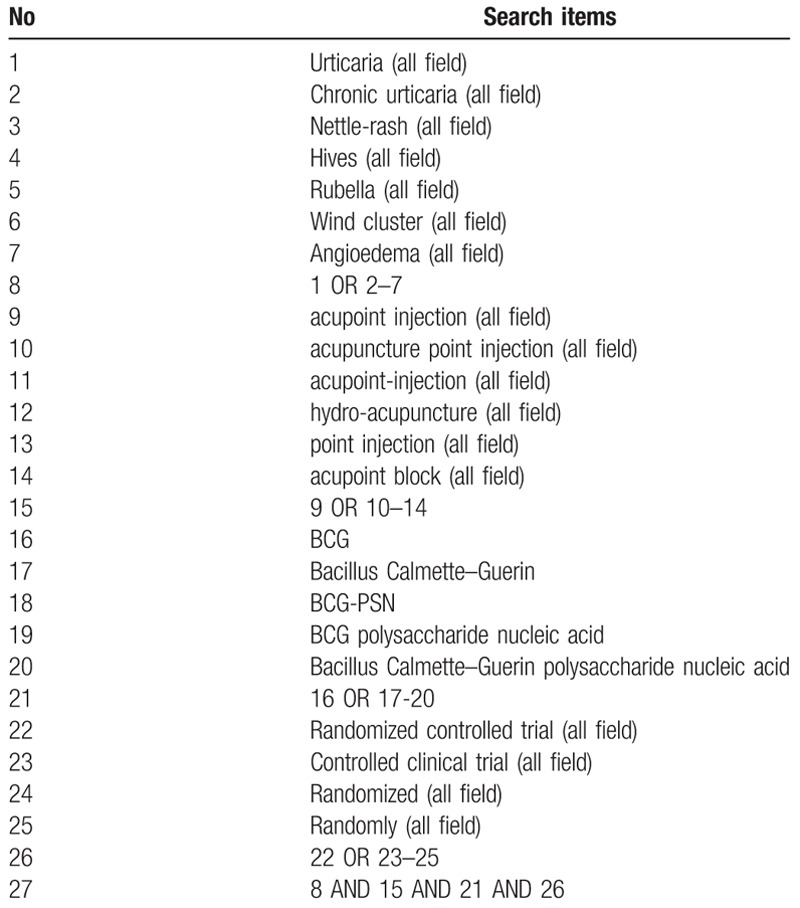
The search strategy used in PubMed.

### 2.4 Criteria for including studies

#### 2.4.1 Types of studies

This article only reviewed the RCT of acupoint injection as the main treatment. The control group included oral medication, non-acupoint, no treatment, placebo, diet and so on. In addition, both Chinese and English publications are subject to language restrictions. RCTs that are not subject to release status will be included, excluding the remaining types of documentation.

#### 2.4.2 Types of participants

Regardless of race, gender, age, and education, in our SR patients must comply with the European Academy of Allergology and Clinical Immunology, the Global Allergy and Asthma European Network, World Allergy Organization (EAACI/GA2LEN/EDF/ WAO) guidelines^[1]^ or the Chinese guidelines for the diagnosis and treatment of urticaria version.^[59]^

#### 2.4.3 Types of interventions and comparisons

Qualified interventions in the trial group were based on sterile syringes. Interventions include acupoint injection of BCG-PSN alone or in combination with other active therapies for CU. If combined with other methods, only the control group with the same intervention measures as the experimental group was included. The trial group such as Acupoint injection BCG-PSN combined with other traditional Chinese therapies (eg, Chinese herb decoction, acupuncture, and other therapies), Acupoint injection BCG-PSN versus non-acupoint injection, will be excluded. The following processes will be compared:

(1) Acupoint injection of BCG-PSN versus no treatment.
(2) Acupoint injection of BCG-PSN versus other active therapies.
(3) Acupoint injection of BCG-PSN plus active therapy versus the same active therapies.
(4) Acupoint injection of BCG-PSN versus placebo or sham Acupoint injection.

#### 2.4.4 Types of outcomes.

##### 2.4.4.1 The primary outcomes

The primary outcome was disease activity control, measured by the urticaria activity score (UAS), urticaria control test (UCT), or other validated symptom scores.

##### 2.4.4.2 The secondary outcomes

(1) Response rate.
(2) Recurrence rate during the follow-up period.
(3) Adverse events.

### 2.5 Data collection and analysis

#### 2.5.1 Selection of studies

**Figure 1:**
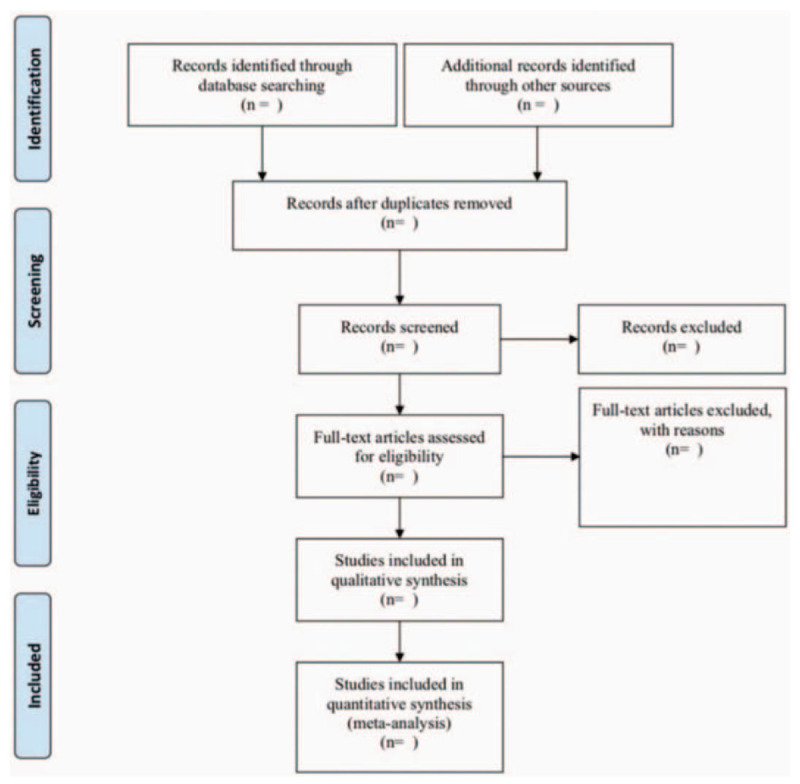
The PRISMA flow diagram of the study selection process. PRISMA=preferred reporting items for systematic reviews and meta-analysis protocols.

References from the search results will be added to the EndNote software (V.X8) document management software and duplicate content will be deleted. Two review authors (XX and LZ) will independently review and screen the titles, abstracts, and keywords of all retrieved studies to confirm eligible trials. The reviewer will receive a full report for further evaluation. Excluded explanations will be recorded in the excel data set. The disagreement between the reviewers (XX and LZ) is still unsolvable by discussion and is determined by a third party'S arbitration (YC).^[41]^ The research flow chart is shown in Figure 1.

#### 2.5.2 Data extraction and management

The 2 review authors (YL and QZ) will extract data independently from the selected report or study and fill out the data extraction form. Extract the following information: general information, participants, methods, interventions, results, results, adverse events, main conclusions, conflicts of interest, ethical approval, etc. When the reported data is insufficient, we will contact the author for more information. In this process, any inconsistencies will be resolved through discussion between the 2 authors or judged by the third author (LW).

### 2.6 Assessment of risk of bias in included studies

The 2 authors (WC and QZ) will assess the study quality by using the checklist developed by the Cochrane Collaboration s bias risk assessment tool which evaluates the presence of potential selection bias (random sequence generation and allocation concealment), performance bias (blinding of investigators and participants), detection bias (blinding of outcome assessors), attrition bias (incomplete outcome data), reporting bias (selective reporting) and possible other sources of bias (funding bias).^[60]^ This review uses L, U, and H as the key to these assessments, where L (low) indicates a lower risk of bias, U (unclear) indicates that the risk of bias is uncertain, and H (high) indicates a higher risk of bias. If inconsistent results appear, the final decisions will be made by the third author (SZ).

### 2.7 Measures of treatment effect

For dichotomous data, risk ratio with 95% confidence interval (CIs) will be used to measure the treatment effect. For continuous data, mean difference (MD) and 95% CIs were used to measure treatment effectiveness.

### 2.8 Dealing with missing data

We will handle missing data in accordance with the guideline stipulated in the Cochrane Handbook for SRs of Intervention. In particular, the following methods will be used:

(1) Contact the corresponding author to request missing data.
(2) Perform analysis of available cases.
(3) Discuss the potential impact of missing data.

### 2.9 Assessment of heterogeneity

Statistical heterogeneity will be assessed for significance with the Cochran *Q* test statistic^[61]^ and quantified with the *I*^2^ value.^[62]^ If statistically significant, the cause will be discussed by narration and the use of subgroups and sensitivity analysis.

### 2.10 Assessment of reporting biases

We will evaluate publication bias using the Egger test and funnel plots.^[63]^

### 2.11 Date synthesis

We will use Review Manager 5.3 for all statistical analyses. The data will be pooled for the meta-analysis when the included studies are sufficiently homogeneous with respect to subjects, interventions, and outcomes. All similar studies will be pooled for a random-effects model to obtain the pooled intervention effect. The pooled intervention effect will be expressed in terms of the MD and 95% of CI if the outcome was reported as a continuous variable. If different scales were used to assess the outcome, the standard MD will be used. If the outcome was measured as a dichotomous variable, we will convert the OR into the standard MD as long as the underlying continuous measure followed an approximately normal distribution.

### 2.12 Subgroup analysis

To resolve some potential problems, we will perform a subgroup analysis. First, we will compare the results of different drug injections at acupoints. Second, we will compare the results of acupoint injection alone with those combined with other active treatments.

### 2.13 Sensitivity analysis

To verify the robustness of the conclusions, sensitivity analyses will be performed to examine the impact of including low-quality studies in the meta-analysis and exclude studies with high or ambiguous bias risk.

### 2.14 Grading the quality of evidence

We will judge the quality of evidence of the results by grading the methods of the recommendations, assessments, developments, and assessments of the working group. Risk of bias, consistency, directness, accuracy, publication bias and additional points were the areas we assessed. The assessment results will be divided into 4 levels: high, moderate, low, or very low.

## 3 Discussion

CU is a disease with high incidence and seriously affects the quality of life and work of patients, but many patients are not satisfied with the current treatment.^[30]^ Acupoint injection of BCG-PSN is an important therapy of integrated Chinese and western medicine. Many studies at home and abroad have shown that it has a considerable effect on CU, and has the advantages of simple operation and low cost,^[48–50]^ but its clinical evidence is insufficient and adverse reactions have been reported. As a drug commonly used in China to treat CU, BCG-PSN has been shown to be effective in many studies,^[53,56]^ while some studies have not found the effectiveness of its combination.^[57]^ This SR will summarize the efficacy and safety of the current evidence and hope to provide convincing evidence for patients and clinicians during the decision-making process.

## Author contributions

**Conceptualization:** Wei Cao

**Data curation:** Xianjun Xiao, Leixiao Zhang, Ying Liu, Lu Wang, Yue Cao, Qianhua Zheng,

**Formal analysis:** Wei Cao, Siyuan Zhou.

**Investigation:** Lu Wang

**Methodology:** Wei Cao, Xianjun Xiao.

**Project administration:** Leixiao Zhang.

**Supervision:** Ying Li.

**Writing – original draft:** Wei Cao.

**Writing – review and editing:** Chunxiao Li, Mingling Chen.

## References

[1] Zuberbier T Aberer W Asero R The EAACI/GA2LEN/EDF/WAO guideline for the defifinition, classifification, diagnosis and management of urticaria. Allergy 2018;73:1393–414. 29336054 10.1111/all.13397

[2] Kaplan AP Diagnosis, pathogenesis, and treatment of chronic spontaneous urticaria. Allergy Asthma Proc 2018;39:184–90. 29669665 10.2500/aap.2018.39.4121

[3] Radonjic-Hoesli S Hofmeier KS Micaletto S Urticaria and angioedema: an update on classification and pathogenesis. Clin Rev Allergy Immunol 2018;54:88–101. 28748365 10.1007/s12016-017-8628-1

[4] Chen Q Zhong H Chen WC Different expression patterns of plasma Th1-, Th2-, Th17- and Th22-related cytokines correlate with serum autoreactivity and allergen sensitivity in chronic spontaneous urticaria. J Eur Acad Dermatol Venereol 2018;32:441–8. 28846158 10.1111/jdv.14541

[5] Berghi NO Immunological mechanisms implicated in the pathogenesis of chronic urticaria and hashimoto thyroiditis. Iran J Allergy Asthma Immunol 2017;16:358–66. 28865416

[6] Takahagi S Mihara S Iwamoto K Coagulation/fibrinolysis and inflammation markers are associated with disease activity in patients with chronic urticaria. Allergy 2010;65:649–56. 19845571 10.1111/j.1398-9995.2009.02222.x

[7] Asero R Tedeschi A Marzano AV Chronic urticaria: a focus on pathogenesis. F1000Res 2017;6:1095. 28751972 10.12688/f1000research.11546.1 PMC5506533

[8] Saini SS Chronic spontaneous urticaria: etiology and pathogenesis. Immunol Allergy Clin North Am 2014;34:33–52. 24262688 10.1016/j.iac.2024.03.002 PMC11218737

[9] Gaig P Olona M Muñoz Lejarazu D Epidemiology of urticaria in Spain. J Investig Allergol Clin Immunol 2004;14:214–20. 15552715

[10] Eun SJ Lee JY Kim D-Y Natural course of new-onset urticaria: results of a 10-year follow-up, nationwide, population-based study. Allergol Int 2019;68:52–8. 29945815 10.1016/j.alit.2018.05.011

[11] Fraser K Robertson L Chronic urticaria and autoimmunity. Skin Therapy Lett 2013;18:5–9. 24305753

[12] Maurer M Weller K Bindslev-Jensen C Unmet clinical needs in chronic spontaneous urticaria. A GA2LEN task force report. Allergy 2011;66:317–30. 21083565 10.1111/j.1398-9995.2010.02496.x

[13] Balp M-M Vietri J Tian H The impact of chronic urticaria from the patient's perspective: a survey in five European countries. Patient 2015;8:551–8. 26476961 10.1007/s40271-015-0145-9 PMC4662955

[14] Confino-Cohen R Chodick G Shalev V Chronic urticaria and autoimmunity: associations found in a large population study. J Allergy Clin Immunol 2012;129:1307–13. 22336078 10.1016/j.jaci.2012.01.043

[15] Najib U Bajwa ZH Ostro MG A retrospective review of clinical presentation, thyroid autoimmunity, laboratory characteristics, and therapies used in patients with chronic idiopathic urticaria. Ann Allergy Asthma Immunol 2009;103:496–501. 20084843 10.1016/S1081-1206(10)60266-9

[16] Nebiolo F Bergia R Bommarito L Effect of arterial hypertension on chronic urticaria duration. Ann Allergy Asthma Immunol 2009;103:407–10. 19927539 10.1016/S1081-1206(10)60360-2

[17] Toubi E Kessel A Avshovich N Clinical and laboratory parameters in predicting chronic urticaria duration: a prospective study of 139 patients. Allergy 2004;59:869–73. 15230821 10.1111/j.1398-9995.2004.00473.x

[18] Hon KL Leung AKC Ng WGG Chronic Urticaria: an overview of treatment and recent patents. Recent Pat Inflamm Allergy Drug Discov 2019;13:27–37. 30924425 10.2174/1872213X13666190328164931 PMC6751347

[19] Zuberbier T Balke M Worm M Epidemiology of urticaria: a representative cross-sectional population survey. Clin Exp Dermatol 2010;35:869–73. 20456386 10.1111/j.1365-2230.2010.03840.x

[20] Maurer M Abuzakouk M Bérard F The burden of chronic spontaneous urticaria is substantial: real-world evidence from ASSURE-CSU. Allergy 2017;72:2005–16. 28543019 10.1111/all.13209 PMC5724512

[21] Weldon DR Quality of life in patients with urticaria. Allergy Asthma Proc 2006;27:96–9. 16724624

[22] Baiardini I Giardini A Pasquali M Quality of life and patients’ satisfaction in chronic urticaria and respiratory allergy. Allergy 2003;58:621–3. 12823121 10.1034/j.1398-9995.2003.00091.x

[23] Kang MJ Kim HS Kim HO The impact of chronic idiopathic urticaria on quality of life in korean patients. Ann Dermatol 2009;21:226–9. 20523794 10.5021/ad.2009.21.3.226 PMC2861226

[24] Sussman G Hébert J Gulliver W Insights and advances in chronic urticaria: a Canadian perspective. Allergy Asthma Clin Immunol 2015;11:7. 25705232 10.1186/s13223-015-0072-2 PMC4336710

[25] Godse K De A Zawar V Consensus statement for the diagnosis and treatment of Urticaria: a 2017 update. Indian J Dermatol 2018;63:2–15. 29527019 10.4103/ijd.IJD_308_17 PMC5838750

[26] Powell RJ Leech SC Till S BSACI guideline for the management of chronic urticaria and angioedema. Clin Exp Allergy 2015;45:547–65. 25711134 10.1111/cea.12494

[27] Shahzad Mustafa S Sánchez-Borges M Chronic Urticaria: comparisons of US, European, and Asian guidelines. Curr Allergy Asthma Rep 2018;18:36. 29796863 10.1007/s11882-018-0789-3

[28] Yu L Buttgereit T Stahl Skov P Immunological effects and potential mechanisms of action of autologous serum therapy in chronic spontaneous urticaria. J Eur Acad Dermatol Venereol 2019;33:1747–54. 31025425 10.1111/jdv.15640

[29] Guillén-Aguinaga S Jáuregui Presa I Aguinaga-Ontoso E Updosing nonsedating antihistamines in patients with chronic spontaneous urticaria: a systematic review and meta-analysis. Br J Dermatol 2016;175:1153–65. 27237730 10.1111/bjd.14768

[30] Kim JK Har D Brown LS Recurrence of chronic urticaria: incidence and associated factors. J Allergy Clin Immunol Pract 2018;6:582–5. 28888844 10.1016/j.jaip.2017.07.012

[31] Sarkar TK Sil A Pal S Effectiveness and safety of levocetirizine 10 mg versus a combination of levocetirizine 5 mg and montelukast 10 mg in chronic urticaria resistant to levocetirizine 5 mg: a double-blind, randomized, controlled trial. Indian J Dermatol Venereol Leprol 2017;83:561–8. 28656910 10.4103/ijdvl.IJDVL_551_16

[32] Simons FER Simons KJ Histamine and H1-antihistamines: celebrating a century of progress. J Allergy Clin Immunol 2011;128:1139–50. 22035879 10.1016/j.jaci.2011.09.005

[33] Saruwatari J Matsunaga M Ikeda K Impact of CYP2D610 on H1-antihistamine-induced hypersomnia. Eur J Clin Pharmacol 2006;62:995–1001. 17089107 10.1007/s00228-006-0210-3

[34] Guillet G Bécherel P-A Pralong P The burden of chronic urticaria: French baseline data from the international real-life AWARE study. Eur J Dermatol 2019;29:49–54. 30827942 10.1684/ejd.2018.3495

[35] Wu KCP Jabbar-Lopez ZK Omalizumab, an Anti-IgE mAb, receives approval for the treatment of chronic idiopathic/spontaneous urticaria. J Invest Dermatol 2015;135:13–5. 25501377 10.1038/jid.2014.362

[36] Cordeiro Moreira AS Rosmaninho Lopes de Soares E Silva MI Use of omalizumab in the treatment of chronic urticaria. Eur Ann Allergy Clin Immunol 2016;48:242–6. 27852430

[37] Metz M Ohanyan T Church MK Omalizumab is an effective and rapidly acting therapy in difficult-to-treat chronic urticaria: a retrospective clinical analysis. J Dermatol Sci 2014;73:57–62. 24060603 10.1016/j.jdermsci.2013.08.011

[38] Delong LK Culler SD Saini SS Annual direct and indirect health care costs of chronic idiopathic urticaria: a cost analysis of 50 nonimmunosuppressed patients. Arch Dermatol 2008;144:35–9. 18209166 10.1001/archdermatol.2007.5

[39] Shi Y Zheng H Zhou S Efficacy and safety of acupuncture for patients with chronic urticaria: study protocol of a randomized, sham-controlled pilot trial. Trials 2019;20:326. 31164178 10.1186/s13063-019-3433-1 PMC6549330

[40] Yao Q Li S Liu X The effectiveness and safety of acupuncture for patients with chronic urticaria: a systematic review. Biomed Res Int 2016;2016:5191729. 27314024 10.1155/2016/5191729 PMC4897793

[41] Xiao X Shi Y Zhang L Cupping for patients with chronic urticaria: a systematic review protocol. Medicine (Baltimore) 2019;98:e17115. 31567949 10.1097/MD.0000000000017115 PMC6756615

[42] Sha T Gao LL Zhang CH An update on acupuncture point injection. QJM 2016;109:639–41. 27083985 10.1093/qjmed/hcw055

[43] Chen YH Wang HP Hong XY Progress of research on acupoint injection therapy mechanism. Shanghai Zhen Jiu Za Zhi 2005;11:47–9.

[44] Wang M Gao Y-H Xu J Zusanli (ST36) acupoint injection for preventing postoperative ileus: a systematic review and meta-analysis of randomized clinical trials. Complement Ther Med 2015;23:469–83. 26051583 10.1016/j.ctim.2015.03.013 PMC4909358

[45] Li MH Guo Y Progress and prospects of research on acupoint injection. Zhenjiu Lin Chuan Za Zhi 2010;26:69–72.

[46] Huang F Xu C Li B [Acupoint injection of BMSCs combined with Chinese herbs for capillary density in ischemic hind limb of diabetes mellitus rats]. Zhongguo Zhen Jiu 2018;38:969–77. 30672183 10.13703/j.0255-2930.2018.09.017

[47] Zheng K-H Wu M-X [Efficacy of acupuncture combined with hydro-acupuncture for transverse processes syndrome of the third lumbar vertebra]. Zhongguo Zhen Jiu 2019;39:28–32. 30672252 10.13703/j.0255-2930.2019.01.006

[48] Yu Y Fan H Cheng Y [Effect of intravenous granisetron combined and acupuncture point injection at PC6 (Neiguan) with 0.9% sodium chlorideon postoperative nausea and vomiting after gynecological laparoscopy]. Zhonghua Yi Xue Za Zhi 2019;99:2606–10. 31510721 10.3760/cma.j.issn.0376-2491.2019.33.010

[49] Xu X-K Jia C-S Wang J-L [Analysis on characteristics and regularities of efficacies of acupoint injection by using data mining technique]. Zhen Ci Yan Jiu 2012;37:155–60. 22764604

[50] Fang Q Li Y Cai M-Y [Efficacy of Ropivacaine injection at acupoints for labor analgesia and its effect on breastfeeding and serum prolactin]. Zhen Ci Yan Jiu 2019;44:434–8. 31368267 10.13702/j.1000-0607.190009

[51] Sun J Hou J Li D Enhancement of HIV-1 DNA vaccine immunogenicity by BCG-PSN, a novel adjuvant. Vaccine 2013;31:472–9. 23174201 10.1016/j.vaccine.2012.11.024

[52] Li N Cao N Niu Y-D Effects of the polysaccharide nucleic acid fraction of bacillus Calmette-Guérin on the production of interleukin-2 and interleukin-10 in the peripheral blood lymphocytes of patients with chronic idiopathic urticaria. Biomed Rep 2013;1:713–8. 24649015 10.3892/br.2013.130 PMC3916996

[53] Chen Z-W Nie X-L Su A-X [Therapeutic effect of BCG polysaccharide nucleic acid injection combined with desloratadine in the treatment of chronic urticaria]. Lin Chuang Pi Fu Ke Za Zhi 2005;5:329.

[54] Jie X-M Gao H Observation on therapeutic effect of BCG polysaccharide nucleic acid injection at acupoint on chronic urticaria. Zhongguo Zhong Xi Yi Jie He Pi Fu Bing Xue Za Zhi 2018;17:247–9.

[55] Yu P Observation on therapeutic effect of BCG polysaccharide nucleic acid injection on chronic urticaria. Xian Dai Zhen Duan Yu Zhi Liao 2015;26:3875–6.

[56] Wang G-J Jing L-H Liu W-B [Observation on therapeutic effect of treating chronic urticaria by acupuncture point injection of ebastin combined with BCG polysaccharide nucleic acid]. Xian Dai Zhong Xi Yi Jie He Za Zhi 2009;18:3299–300.

[57] Yan S Liu R Mao M Therapeutic effect of Bacillus Calmette-Guerin polysaccharide nucleic acid on mast cell at the transcriptional level. PeerJ 2019;7:e7404. 31497384 10.7717/peerj.7404 PMC6708377

[58] Moher D Shamseer L Clarke M Preferred reporting items for systematic review and meta-analysis protocols (PRISMA-P) 2015 statement. Syst Rev 2015;4:1. 25554246 10.1186/2046-4053-4-1 PMC4320440

[59] IgoCSo D Chinese guidelines for the diagnosis and treatment of urticaria version. Chin J Dermatol 2014;47:514–6.

[60] Yao J Chen L Zhang L Effect of auriculotherapy and intervention types on weight control: a systematic review and meta-analysis protocol. Medicine (Baltimore) 2019;98:e16959. 31441898 10.1097/MD.0000000000016959 PMC6716699

[61] Zhang L Xiao X Hui R Autologous whole-blood or autologous serum acupoint injection therapy for chronic urticaria: a systematic review protocol. Medicine (Baltimore) 2019;98:e16127. 31232963 10.1097/MD.0000000000016127 PMC6636973

[62] Qin D Yue L Xue B Pharmacological treatments for patients with irritable bowel syndrome: an umbrella review of systematic reviews and meta-analyses. Medicine (Baltimore) 2019;98:e15920. 31393342 10.1097/MD.0000000000015920 PMC6709044

[63] Egger M Davey Smith G Schneider M Bias in meta-analysis detected by a simple, graphical test. BMJ 1997;315:629–34. 9310563 10.1136/bmj.315.7109.629 PMC2127453

